# A glance at structural biology in advancing rice blast fungus research

**DOI:** 10.1080/21505594.2024.2403566

**Published:** 2024-09-16

**Authors:** Jongyi Yan, Lin Li, Jiandong Bao, Jiaoyu Wang, Xiaohong Liu, Fucheng Lin, Xueming Zhu

**Affiliations:** a State Key Laboratory for Managing Biotic and Chemical Threats to the Quality and Safety of Agro-Products, Zhejiang Provincial Key Laboratory of Agricultural Microbiomics, Key Laboratory of Agricultural Microbiome (MARA), Institute of Plant Protection and Microbiology, Zhejiang Academy of Agricultural Sciences, Hangzhou, Zhejiang, China; b State Key Laboratory for Managing Biotic and Chemical Threats to the Quality and Safety of Agro-products, Zhejiang Provincial Key Laboratory of Agricultural Microbiomics, Key Laboratory of Agricultural Microbiome (MARA), Institute of Biotechnology, Zhejiang University, Hangzhou, Zhejiang, China; c Xianghu Laboratory, Hangzhou, Xianghu, China

**Keywords:** *Magnaporthe oryzae*, protein structure, drug targets, structure-based drug design

## Abstract

The filamentous fungus *Magnaporthe oryzae* is widely recognized as a notorious plant pathogen responsible for causing rice blasts. With rapid advancements in molecular biology technologies, numerous regulatory mechanisms have been thoroughly investigated. However, most recent studies have predominantly focused on infection-related pathways or host defence mechanisms, which may be insufficient for developing novel structure-based prevention strategies. A substantial body of literature has utilized cryo-electron microscopy and X-ray diffraction to explore the relationships between functional components, shedding light on the identification of potential drug targets. Owing to the complexity of protein extraction and stochastic nature of crystallization, obtaining high-quality structures remains a significant challenge for the scientific community. Emerging computational tools such as AlphaFold for structural prediction, docking for interaction analysis, and molecular dynamics simulations to replicate in vivo conditions provide novel avenues for overcoming these challenges. In this review, we aim to consolidate the structural biological advancements in *M. oryzae*, drawing upon mature experimental experiences from other species such as *Saccharomyces cerevisiae* and mammals. We aim to explore the potential of protein construction to address the invasion and proliferation of *M. oryzae*, with the goal of identifying new drug targets and designing small-molecule compounds to manage this disease.

## Introduction

Structural biology is a multidisciplinary field that focuses on the study of three-dimensional structures of biological molecules and their complexes, as well as the relationships between structure and function. It encompasses a wide range of experimental and computational techniques aimed at elucidating the atomic-level details of biomolecules, including proteins, nucleic acids, lipids, and carbohydrates. In agriculture, structural biology has provided crucial insights into the molecular mechanisms of pattern recognition receptors (PRRs), nucleotide binding leucine-rich repeat proteins (NLRs) and effectors [1]. By elucidating the structures of key immune receptors, signalling components, and pathogen effectors, structural biology contributes to a deeper understanding of plant–pathogen interactions and the development of strategies for crop protection and disease resistance.

Thus far, research into the structural biology of plant pathogenic fungi has become a useful tool for understanding the mechanisms underlying their pathogenicity mechanisms, host interactions, and for developing effective strategies for disease control. Give some examples about structural biology applications, identifying a single phenamacril-binding residue in phenamacril-sensitive myosins from *Fusarium graminearum* advances our understanding of phenamacril selectivity and resistance mechanisms in fungal pathogens [2]. The discovery of the effector SIB1 in *Colletotrichum*, with its unique structure and ability to suppress PAMP-triggered immunity (PTI), significantly advances our understanding of fungal pathogenicity and host–pathogen interactions [3]. Structural analyses reveal that BcBOT2 in *Botrytis cinerea* undergoes dynamic conformational transitions from an open to a closed state upon substrate binding [4]. In summary, structural biology plays a pivotal role in understanding the interactions between plant pathogenic fungi and their hosts, facilitating the discovery of new drugs and unravelling the functions of critical proteins involved in fungal metabolism, cell wall synthesis, signalling cascades, and virulence.

**Figure 1.**
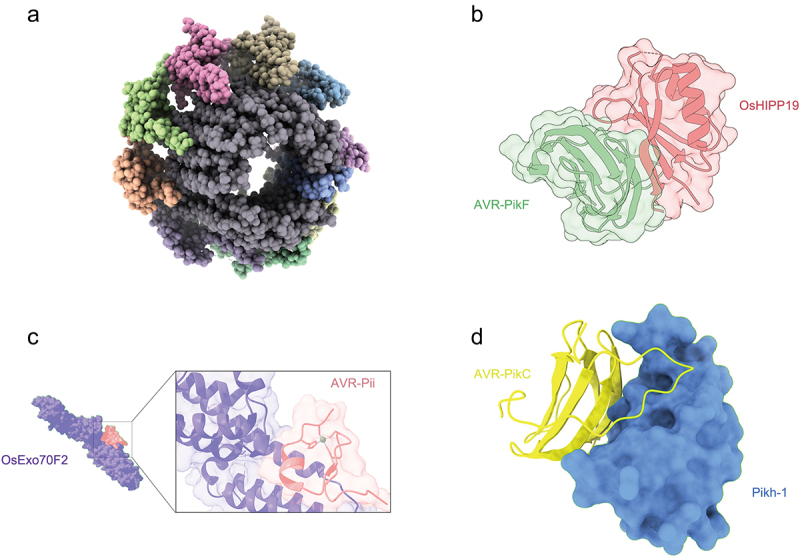
Protein complex structure determined by X-ray diffraction and cryo-electron microscopy. (a) YAR027W and YAR028W interacting with the c subunits of the V-ATPase complex (PDB ID: 8eav). (b) The crystal structure of AVR-PikF (green) with the HMA domain of OsHIPP19 (red). (c) The crystal structure of OsExo70F2 (violet) in complex with AVR-Pii (red). (d) AVR-PikC with the HMA domain of pikh-1.

*Magnaporthe oryzae* is an ascomycete fungus that exists in the haploid state. It exhibits growth through branching hyphae and spreads via tear-shaped, three-celled asexual conidia [5]. These conidia were formed on aerial hyphae. *M. oryzae* is also a plant pathogenic fungus that causes rice blast disease, which is one of the most devastating diseases affecting rice crops worldwide [6]. It also infects other cereal crops such as wheat, barley, and millet. In rice blast fungus research, structural biology is primarily directed towards comprehending the mechanisms by which key proteins function within the pathway, illuminating host–pathogen interactions, and pioneering the development of innovative antifungal strategies [7]. In recent years, there has been an increase in the identification of effectors within the interaction network between rice and *M. oryzae*. Specifically, 40 *AVR* genes have been reported and subjected to genetic analysis [8]. Advancements in computational structure prediction and structural biology techniques have facilitated more accurate descriptions of the functions of fungal effectors. Understanding the structural biology of *M. oryzae* is crucial for developing effective strategies to combat rice blast disease and to mitigate its impact on global food security. Using structural biology methodologies, primarily X-ray crystallography, and Nuclear Magnetic Resonance (NMR), researchers have delved into the intricate details of protein structure. This provided critical insights into how these proteins operate within the fungal pathway, shedding light on their roles and interactions. With the broad use of cryo-electron microscopy (cryo-EM) technology, scientists can visualize the structures of macromolecules and complexes at near-atomic resolution without the need for crystallization. Moreover, cryo-EM has been instrumental in revealing the polymer structure of yeast organelles, such as VO (Vma12-22), which reveals how Vma12-22 and Vma21 coordinate in assembling (Figure 1a) [9]. This advancement made in yeast structural biology through cryo-EM could be widely applied to study rice blast fungus in the future.

In this review, we consolidate the structural biology advancements in *M. oryzae*, leveraging established experimental techniques from other species such as yeasts and mammals. Our goal is to explore the potential of protein engineering to analyse pathogen–host interactions at the molecular level, facilitate structure-based drug screening, and elucidate the in vivo functions of these critical proteins.

### The structural insights of effector-interactor complex in *M. oryzae*

In plant pathogen infection process, several recent studies have demonstrated that PTI and effector-triggered immunity (ETI) are intertwined and responses to pathogen infection [10–12]. The first is initiated by PRRs located on the cell surface, resulting in PTI and another is activated by NLRs, leading to ETI. PTI and ETI are not entirely separate pathways but are interconnected. Successful activation of ETI often depends on components initially involved in PTI [13]. ETI responses are often more robust and sustained compared to PTI. While PTI provides a general defence response, ETI amplifies this response by targeting specific pathogen effectors, leading to stronger and more specific immune reactions [14]. Understanding how ETI contributes to pathogen resistance depends on the intricate relationship between the effectors and their corresponding receptors. Over the past five years, research on the structure of *M. oryzae* has predominantly focused on elucidating the interactions between Avr effectors and their corresponding receptors in rice. Advances in structural biology have played a crucial role in the study of effectors and NLRs in *M. oryzae*, thereby uncovering increasingly intricate interaction mechanisms.

NLRs typically have a multi-domain structure, featuring a central nucleotide-binding (NB-ARC) domain and C-terminal LRR regions. Additionally, they commonly include N-terminal coiled-coil (CC) or TOLL/interleukin-1 receptor (TIR) domains to confer resistance [15]. To date, approximately 100 NLRs in rice have been identified as conferring resistance to strains of *M. oryzae* [15], but only a few of their associations with effectors have been clearly elucidated. The NMR structures of Avr-Piz-t and Avr-Pia suggest that these proteins employ a shared mechanism for interacting with host plant target proteins [16,17].

The MAX-effectors (*Magnaporthe* Avrs and ToxB like) represent a well-known protein family exhibiting sequence-unrelated yet structurally conserved characteristics, serving as a paradigm for fungal effector diversity [18,19]. Comparing the NMR structures of Avr1-CO39 and Avr-Pia with those of AvrPiz-t and ToxB, which are homologs of Avr1-CO39 and Avr-Pia, a shared architectural framework has been observed, despite the absence of sequence consensus [20]. Disulfide bond plays a crucial role in stabilizing the structure of MAX effectors; however, in Zhang’s study involved mutating key amino acids located in the positive-charge patch on the surface of Avr-Pib (K29A/E, K30A/E, R50A/E, K52A/E, and K70A/E), hydrophobic residues replace cysteine and contribute to structural stability [21]. The mutant failed to be recognized by rice carrying Pib, indicating that surface charge distribution is a key factor contributing to recognition specificity compared with other MAX effectors. The Heavy Metal-Associated domain of Pikp (Pikp^HMA^) has been shown to interact with Avr-PikD, and structural analysis revealed that the binding interfaces between Avr-PikD and Pikp^HMA^ could be categorized into three primary sites, each contributing to the stabilization of the complex [22]. Beyond this, His46, embedded in a pocket on the Pikp^HMA^ surface, aids in forming hydrogen bond or salt bridge interactions. The complex structure of the Pikp^HMA^/Avr-PikD provides a foundation for introducing designed mutations to modify protein interactions in both yeast and in vitro systems, thereby perturbing effector-mediated resistance. NLR immune receptors interact specifically with their corresponding effectors, thereby initiating plant defence mechanisms. Several studies have indicated an association between Pikp and Avr-PikD [22,23]; however, Varden et al. revealed that NLR immune receptor can bind diverse pathogen effectors through an integrated domain. Pikp can interact with both its designated counterpart, Avr-PikD, and the mismatched effector Avr-Pia [24]. Their study empirically substantiated the resistance of rice plants bearing Pikp against *M. oryzae*. The disparate affinity between Pikp and Avr-PikA compared to Avr-PikD can be attributed to two factors. Firstly, the half size interface of Avr-PikA, contributing to weaker binding. Additionally, the limited hydrogen bond/salt bridge interactions within the Pikp^HMA^/Avr-PikA complex. In contrast to the previous binding of Avr-Pik to Pik-1^HMA^, OsHIPP19^HMA^ interacts with Avr-PikF/C, suggesting that the HMA domain can be utilized to broaden resistance to Avr-Pik variants (Figure 1b). Surface Plasmon Resonance (SPR) results show that Avr-Pik binds to OsHIPP19^HMA^ with greater affinity than to integrated Pik-1 HMA domains, and this increased binding is due to structural differences in the interacting interfaces [25].

Most Avr-effectors adopt a MAX-effector fold to recognize receptors, and a distant zinc-finger fold that has not been previously reported for any other plant pathogen effectors was found by De la Concepcion et al. [26]. The binding interface showed that Avr-Pii associates with OsExo70F2 through a hydrophobic pocket (Figure 1c). And the Tyr64Arg and Phe65Glu mutation results in a lack of binding and further abrogates Pii-mediated resistance to rice blast. Lahfa’s presentation of NMR structures for the recently identified putative MAX effectors, MAX47/60/67 indicated a conserved β-strands in all three proteins. This finding aligns with the typical β-sandwich architecture commonly observed in MAX effectors, which aids in predicting the sequences and structures of other MAX effectors [19]. The NMR structure of type III CVNH/LysM revealed two distinct and functionally independent domains (CVNH and LysM). This structural arrangement mirrors functional autonomy, highlighting a fascinating example of protein evolution [27]. MoHrip2 acts as a protein elicitor that stimulates immune response in tobacco plants. By analysing its structure, researchers have identified 14 amino acids that play critical roles in both triggering the hypersensitive response and conferring disease resistance [28]. Structural and enzymatic analyses of the Nudix effector family have revealed their selective ability to digest inositol pyrophosphates [29]. Structural biology analysis not only facilitates the elucidation of molecular structures but also empowers the precise design of genes, thereby enabling the modulation of corresponding protein interactions. This advancement has the potential to make engineering disease resistance a tangible and achievable goal. Previous studies on NLR immune receptors have highlighted that a specific decoy domain plays a crucial role in defence mechanisms [30,31]. However, for adapting to the environment, sometimes one interaction site cannot recognize the effector exactly. Avr-Pia associates with additional site besides RGTX1 domain with β-strands 2/3 and residues R23, F24, E56, E58 [32]. However, the reduction in these sites does not impair recognition. Resistance mediated by RGA5 exhibits a high degree of resilience to effector mutations. Furthermore, β-strands 2/3 and residues R23, F24, E56, E58 of Avr-Pia protein was identified as candidate interaction surfaces, supplementing the functional association [32].

Utilizing structural information to guide rational engineering of NLRs is a promising strategy for modifying their recognition spectra. Recent studies demonstrating the engineering of an HMA domain within Pikp-1, incorporating residues from the HMA domain of the Pikm1 allele, exhibited an augmented capacity to discern related MAX effectors – Avr-PikA/D/E [33]. Within this context, the researchers have engineered an NLR receptor, RGA5^HMA2^, conferring immunity against the non-corresponding effector Avr-Pib [34]. It was also established that the HMA domain of RGA5 can recognize two sequence-unrelated effectors, Avr-Pia, and Avr1-CO39 [35]. These meticulously designed outcomes demonstrated the successful establishment of immune capabilities, thereby contributing valuable insights into plant–pathogen interactions. Another example, RGA5^HMA5^, imparts complete resistance against *M. oryzae* strains expressing the noncorresponding effector Avr-PikD [36]. Additionally, the C-terminal lysine-rich stretch trailing the HMA ID in RGA5^HMA5^ plays a crucial role in both recognizing MAX effectors and activating RGA4-dependent rice immunity. Engineering of NLRs and their associated immune domains focused on enhancing the ability to recognize and respond to pathogens has become a significant area of research in plant and animal immunity. The Pikp-1^NK-KE^ variant, derived from the engineering of the Pikp-1 HMA ID, demonstrates an extended recognition spectrum targeting various Avr-Pik alleles [37]. De la Concepcion et al. employed a structure-guided approach by introducing Pikp^NK-KE^ (Asn261Lys, Lys262Glu) mutation, leading to a interface resembling that of Pikm [26]. Consequently, the mutation elicited a Pikm-like response to Avr-PikE/A. NLRs can also serve as scaffolds for nanobody (single-domain antibody fragment) fusion capable of binding fluorescent proteins (FPs). These fusion proteins induce immune responses when the corresponding FPs are present. Therefore, plants expressing these recombinant proteins could activate an immune response upon the introduction of fluorescent proteins, thereby expanding the range of pathogens that plants can detect [38]. Understanding the interface-binding sites between Avr proteins and their receptors helps design drugs that can specifically target these proteins to interfere with the functions of the pathogen, reducing resistance caused by mutations and minimizing off-target effects [13,39]. In addition to changing functional fragments could extend recognition, single amino acid polymorphisms can also increase binding affinity. The Asn261Lys polymorphism in Pikh^HMA^ extends the recognition of Avr-Pik variants in a manner similar to Pikm [40] (Figure 1d). Additionally, structure-guided mutagenesis screened the virulence-related sites (Avr2^T53^ and Avr2^T145^) as well as the mutants (Avr2^T53R^, Avr2^T145K^, and Avr2^T145E^) maintained their ability to interact with the host target in a manner akin to the wild-type protein [41]. Confirming the structural interactions between host and pathogen enhances the precision of detecting specific effectors. This enables early identification of infections and timely prevention and control. In the future, structural insights will help produce resistant plant varieties that more effectively counteract effectors and introduce specific Avr genes to boost plant defence against pathogens. Fundamentally, the advancement of Artificial Intelligence (AI) structure prediction relies heavily on the support of extensive high-quality protein structure data. By comparing common and specific structures, it can provide more accurate predictions for proteins that are difficult to analyse.

The significance of structural biology of *M. oryzae* effectors lies in elucidating the three-dimensional structures of proteins and understanding their interactions with host receptors. By determining the atomic-level structures of effectors and their targets, structural biology can provide insights into their functions, mechanisms of action, and evolutionary relationships. This knowledge is crucial for understanding how *M. oryzae* pathogens manipulate host cells and evade immune responses and can ultimately inform the development of new strategies for disease control and breeding. Overall, structural biology plays a vital role in unravelling the molecular basis of fungal pathogenesis and in advancing efforts to combat fungal diseases.

### Advanced green strategies for the prevention and control of rice blast based on protein structure

Structure-based drug design (SBDD) for chemical molecule screening is a wide area of identification for the selective inhibitors of a target of interest. Several successful applications have been reported, particularly in the field of virtual screening combined with computer-aided molecular docking. Candidate compounds were synthesized and experimentally evaluated using ADMET (Absorption, Distribution, Metabolism, Excretion, and Toxicity). Since bioactive small molecules have been discovered, the structure of a ligand-receptor complex can be obtained during crystallization. After that, the interaction site and functional group within the binding interface could be identified exactly, which would expand the spectrum of drugs. This process begins with new steps to incorporate molecular modifications with the potential to increase the affinity of new ligands for the binding site. For the past few years, SBDD has played an indispensable role in the treatment of a wide variety of diseases in different species. The co-crystal structures of sulfatinib, a potent target FGFR inhibitor, binding with FGFR1 and CSF-1 R demonstrate the mechanism of inhibitor targeting and kinase specificity [42]. Furthermore, the structure of CSF-1 R/FGFR1 with sulfatinib could be a foundation for optimizing FGFR inhibitors with increased potency against CSF-1 R. SBDD has been extensively employed in the human drug discovery pipeline. Amprenavir, identified as a potential inhibitor of HIV protease, was discovered through protein modelling and molecular dynamics (MD) simulations [43]. Other successful cases of SBDD include raltitrexed (a thymidylate synthase inhibitor) [44], Norfloxacin (an antibiotic) [45] and Carbonic anhydrase [46]. Employing structural insights into ASK1 and deconstructing established inhibitors, researchers devised a groundbreaking ASK1 inhibitor named compound 2 (GS-4997, Gilead Sciences). This new compound demonstrated remarkable potency and oral bioavailability [47].

**Figure 2.**
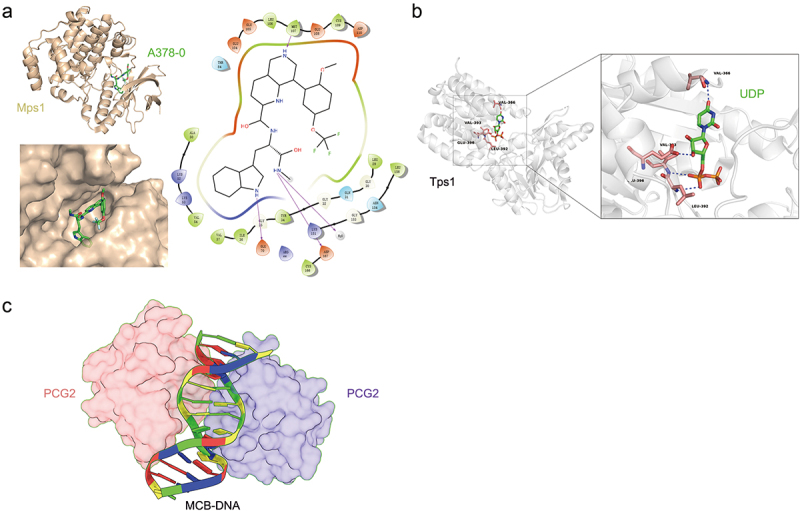
Schematic diagram of protein-molecule and nucleic acid complex structure. (a) Structure of Tps1 (grey)/udp (green) complex. (b) Overall structure of the Mps1 (yellow)/A378-0 (green) complex. (c) The asymmetric structure of the complex consists of two monomers of PCG2–DBD, monomer-a, B (pink, violet) and one molecule of MCB – DNA.

High-throughput structure-based drug design (HT-SBDD) has the potential to enhance the effectiveness of High-Throughput Screening (HTS) methods [48]. Kong et al. adopted a DNA-encoded compound library (DEL) for Mps1 and successfully identified A378–0 as an inhibitor of appressorium penetration and invasive growth [49]. Moreover, the crystal structure of the Mps1/A378-0 complex combined with bioactivity evaluation confirmed that A378–0 inhibited *M. oryzae* infection by specifically targeting Mps1 (Figure 2a). Wu et al. identified SP-141 as a potential lead compound targeting Trs85 to prevent rice blasts using virtual screening [50]. Furthermore, bioactive assays showed that SP-141 is involved in the infection and macroautophagy of *M. oryzae* and is also a broad-spectrum fungicide for rice blasts and other fungi. Compound a2 was synthesized to inhibit laccase activity, which exerts antifungal effects by inhibiting the growth of pathogenic mycelium. Sun et al. proposed that the addition of hydrogen bonds within Asn264 and Pro394 contribute to the binding affinity based on docking analysis, which plays crucial roles in the interaction [51]. Based on the a2-laccase docking interface, compound m14 was designed from thirty-eight novel derivatives after a series of optimizations, including the introduction of morpholine and piperazine to form hydrogen bond [52]. The interaction between carpropamid and scytalone dehydratase (SDH) appears to be elucidated through crystal structure analysis. The specific groups within carpropamid, including the chloride atom, (chlorophenyl) ethyl group, and carboxamide group, were observed to form contacts with SDH. These interactions are likely responsible for the tight binding observed between carpropamid and SDH [53]. To discover novel SDH inhibitors (SDHIs), the binding modes, and interactions of compound 5 l and fluxapyroxad with SDH were compared and their interaction modes were found to be similar, including hydrogen bonding and hydrophobic interactions. However, residues of SDH involved in binding with compound 5 l exhibited a lower binding free energy than when binding with fluxapyroxad, which suggests that 5 l forms stronger associations [54].

Recently, dual-interaction targeting has become a valuable strategy in drug design aimed at enhancing specificity. By targeting multiple interaction sites on a protein or enzyme, a drug can achieve greater selectivity for its intended target while minimizing off-target effects. Based on the crystal structure of trehalose-6-phosphate synthase MoTps1 (Figure 2b), virtual screening targeting the trehalose-6-phosphate phosphatase TPS/TPP pathway discovered A1–4, with dual specific association to the substrate pockets of TPS/TPP, which led to a stronger binding affinity to MoTps1 (26.2 μM) and MoTps2-TPP (59.8 μM) [55]. These double interactions with MoTps1 and MoTps2-TPP confer A1–4 a higher inhibition of the enzyme activity as well as a lower EC50 for inhibiting spore germination compared to other candidates. Additionally, TPS and TPP cannot be synthesized in animals due to the absence of trehalose, and the non-specific binding of A1–4 hits could be decreased, confirming the safety of further drug promotion. MoErs1, a specific target effector that shares no sequence similarity with other species including fungi, can interact with OsRD21 from rice and form a flexible and narrow area with more hydrophobic amino acid residues. The double-protein docking pocket makes drug binding more specific. Based on the structure and function of MoErs1, Liu et al. used diaryl ether as a skeleton because of its molecular flexibility and further designed FY21001 by adding hydroxyl and ester groups to form hydrogen bonds and hydrophobic interactions. Consequently, FY21001 effectively prevents rice blast [56]. The binding pocket of FY21001 was formed by the interaction of MoErs1 and OsRD1; hence, the affinity was reduced by FY21001 in a dose-dependent manner.

These studies represent significant advancements in the identification and development of compounds targeting key proteins involved in the pathogenesis of *M. oryzae* based on the receptor protein structure, offering potential avenues for the control of rice blast disease. While SBDD is still in its early developmental phases, the rise of heavily automated HT-SBDD methods is poised to be instrumental in targeting numerous proteins that remain unexplored by drugs, especially those with high-resolution structures. Despite the potential of employing various computational techniques to mitigate the uncertainty in drug candidate identification, experimental validation remains crucial. Therefore, traditional experimental validation approaches must be employed alongside novel SBDD protocols to substantiate any findings derived from SBDD.

Beyond SBDD, gaining structural insights can significantly clarify the breeding directions. By elucidating the three-dimensional architecture of protein–protein interactions or protein-ligand complexes, structural studies provide invaluable information guiding the selection of breeding targets and the design of novel traits in agricultural crops. Chandrakanth et al. modelled structures of eight blast resistance proteins in silico and illustrated conserved NB-ARC and LRR binding domain [57], which lay the foundation for future studies on the interaction mechanism between Avr effector proteins and resistance proteins. Binding between Pi54 and AvrPi54 is crucial for the development of blast disease. Through alignments of the LRR region from Pi54 proteins and molecular dynamics of the Pi54-Avr-Pi54 interaction, 15 resistant Pi54 proteins were determined with lower binding free energy [58]. Hence, these alleles harbour greater potential than the original resistance allele Pi54^tetep^ and represent promising candidates for in future rice blast resistance breeding programs.

Consequently, structural biology contributes to every stage of anti-*M.oryzae* drug discovery process, from target identification and validation to lead optimization and mechanism of action studies. This provides essential insights into the molecular basis of fungal infections and facilitates the development of more effective and selective antifungal therapies.

### Characterizing protein structures is instrumental in understanding fungal development and morphogenesis

Structural biology sheds light on the molecular basis of fungal development and morphogenesis, including spore formation, appressorium development, and hyphal growth. By visualizing the architecture of key regulatory proteins and complexes involved in these processes, structural studies can provide insights into the mechanisms underlying fungal pathogenesis. The structural characterization of the monomeric KS domain offers valuable insights into its unique architecture. By elucidating the three-dimensional structure of the unique monomeric KS domain, Yun et al. identified the His-322 residue and found that the lack of a helical structure due to the presence of His-322 leads to expansion of the substrate-binding pocket, enabling the acceptance of more bulky substrates containing amino acids [59]. Many cell activities occur along with structural changes; thus, probing into structural differences is important for exploring the dynamic process of every component action and specific drug design. Tps1 catalyzes UDP-glucose (UDPG) and glucose-6-phosphate (G6P) to form T6P (Trehalose-6-phosphate), and plays a crucial role in plant infection by *M. oryzae*. Wang et al. got the crystal structure of MoTps1/UDP/T6P complex and demonstrated its open to close transition, which demonstrates after associating with G6P, the “shift region” of Tps1 mover into the catalytic site and leads to the Tps1 and the gap between the G6P and UDPG close fully to allow the nucleophilic reaction and transference of the glucose group of UDPG to G6P to begin [60]. MnLOX and FeLOX exhibit structural similarities and catalytic mechanisms common to lipoxygenases, whereas variations in metal ion coordination, substrate tethering by Arg-525, and the presence of conserved Phe residues near the catalytic centre likely underlie their unique features and functions [61].

The interaction between proteins and DNA is key to cell division and is also the premise to initiate gene transcription. Protein-DNA interactions mainly involve histones, transcription factors, DNA methylases, and chromatin remodelling complexes [62]. The MBP1 family proteins are the subunits of MBF cell-cycle transcription factor complexes, which are responsible for DNA binding [63]. The structure of the PCG2–DBD – DNA complex was analysed by crystallization, which uncovered the DNA-binding model of MBP1 homologue PCG2 is unusual compared to the previously confirmed wHTH proteins DNA-binding domain [64]. PCG2 interacted with the core region CGCG of MCB – DNA through two recognition substituents Q82 and Q89 within the wing (Figure 2c). Unlike most wHTH structures, in which helix B is not the main mediator of DNA binding, the wing of the wHTH domain in the PCG2 complex binds to the minor groove and forms the majority of interactions at the centre of the protein – DNA interface. PC4-like proteins are a group of single-stranded DNA (ssDNA) binding proteins known for their roles in transcription regulation, DNA replication, and repair processes. One of the key features of these proteins is their ability to bind ssDNA with high affinity and specificity. The phosphate-mediated conformational changes in ssDNA binding mode including β-surface of Lys84, the presence of positive electron density between Lys84 residue and DNA, and a newly discovered 2dT-PO4-K84 interaction, are pivotal for the function of PC4-like proteins [65]. Previous studies demonstrated that tryptophan/tyrosine substitution can have large effects on the protein function by comparing structures of the DNA complexes of the PC4 W89Y mutant and MoSub1, PC4 ortholog of *M. oryzae*, Y74 of MoSub1 or W89Y PC4 mutant maintain the interaction with one DNA base; a possible explanation is that the presence of the tyrosine phenolic oxygen is unfavourable for association with a second nucleotide. Therefore, W89 of PC4 and Y74 of MoSub1 direct the mode of the DNA–protein interaction and the two constructions of DNA observed in these DNA complexes might represent differences in strategy, which is used by PC4 and Sub1 to unravel or bind DNA [66]. Cell division cycle 5 (Cdc5) is a highly functionally and structurally conserved eukaryotic protein that participates in diverse molecular processes. By structural analysis of splicesome by Cryo-EM and MoCdc5-DBD by crystallization, Wang et al. suggested that Cdc5-DBD regulates development through two different nucleic acid-binding surfaces, one for DNA and another for RNA [67]. K100 and R31 play key roles in DNA and RNA interactions, respectively. This finding suggests that Cdc5-DBD possesses a dual role in nucleic acid regulation, implying that Cdc5 is involved in coordinating processes such as DNA replication, repair, and transcription, as well as RNA processing and splicing.

Protein structure plays a fundamental role in determining protein function, and this principle is significant for understanding *M. oryzae*. Structural biological techniques are indispensable tools for unravelling fungal mechanisms at the molecular level. By providing detailed insights into protein structures, interactions, and functions, structural biology has accelerated research on fungal biology, pathogenesis, and antifungal drug discovery, with broad implications for human health, agriculture, and environmental sustainability.

## Conclusions and perspectives

Structural biology serves as a powerful tool for unravelling the molecular complexities of fungal biology and pathogenesis, driving the development of functional proteins characterizations, understanding the molecular basis of host-pathogen recognition, facilitating drug discovery to combat fungal infections, and elucidating the evolutionary relationships between *M. oryzae* and its adaptation to diverse ecological niches. Structural investigations should extend beyond drug development and host–effector interactions; they should elucidate the intricate relationship between complex structures and functions. Cryo-EM facilitates this exploration by leveraging structural disparities to elucidate distinctions among various species. With AlphaFold2/3 predicting the three-dimensional configurations of proteins from amino acid sequences with unparalleled atomic-level precision, establishing the relationship between the structure and function of many proteins, which are otherwise difficult to obtain structurally, becomes more accessible. Ultimately, these studies will contribute to mitigating the impact of fungal infections on human health and agriculture.

## Data Availability

Data availability were not applicable to this study. No new datasets were generated in this study, and the data cited in this review were obtained from published articles.
